# Parents’ experiences of condition management in children born with esophageal atresia-tracheoesophageal fistula during their early childhood

**DOI:** 10.1186/s13023-026-04288-4

**Published:** 2026-02-27

**Authors:** Michaela Dellenmark-Blom, Lianne Cole, Rosella Micalizzi, John Bennett, Kaylee Woods, Lauren Cardoni, Leah Frain, Abdimajid Mohamed, Jessica Yasuda, Peter Ngo, Anke Widenmann, Graham Slater, Benjamin Zendejas

**Affiliations:** 1 Department of Pediatrics, Institute of Clinical Sciences, University of Gothenburg, Gothenburg, 416 85 Sweden; 2 Department of Pediatric Surgery, Queen Silvia Children’s Hospital, Sahlgrenska University Hospital, Gothenburg, 416 85 Sweden; 3 Esophageal and Airway Treatment Center, Department of Pediatric General Surgery, Boston Children’s Hospital, Harvard Medical School, Boston, MA USA; 4 Division of Gastroenterology, Hepatology and Nutrition, Boston Children’s Hospital, Boston, MA USA; 5 EAT (Esophageal Atresia Global Support Groups), Sommerrainstr. 61, 70374 Stuttgart, Germany

**Keywords:** Esophageal atresia, Rare disease, Condition management, Caregiver, Tracheobronchomalacia

## Abstract

**Background:**

Esophageal atresia-tracheoesophageal fistula (EA-TEF) is a rare congenital malformation of the esophagus and airways. Following surgical repair, children commonly face ongoing aerodigestive morbidity. To address the limited research in this area, this study explored parents’ experiences managing EA-TEF during early childhood, a time marked by high morbidity, and evolving parenting roles.

**Method:**

As part of a larger project, five standardized focus groups were conducted with 22 parents of children aged 6 months to 7 years treated at a quaternary pediatric surgical center in U.S. Discussions were audio-recorded, transcribed, and analyzed using inductive manifest content analysis.

**Results:**

The 22 parents made 629 unique statements about managing their child’s condition. Two themes emerged. The first theme described parents’ experiences of internal day-to-day responsibilities (303 statements) with two categories; Taking responsibility for the child’s health needs (213 statements) and Challenges and possibilities in understanding the child’s condition/symptoms (90 statements). The second theme involved engaging with healthcare and community systems to support their child’s health and development (326 statements), with three categories; Navigating and finding health care and community-based support programs (214 statements); Managing the child’s transition into contexts outside home: babysitters, daycare, and school (74 statements), and Defining the role of patient/peer support (38 statements).

**Conclusions:**

Parents of children with EA-TEF describe facing significant and ongoing responsibilities in managing their child’s aerodigestive health, while navigating complex healthcare, educational and patient/peer support to ensure their children received adequate support. These findings highlight the need for timely, family-centered healthcare programs that address both medical and psychosocial challenges in early childhood.

**Supplementary Information:**

The online version contains supplementary material available at 10.1186/s13023-026-04288-4.

## Background

Esophageal atresia (EA) occurs in approximately 2.4 per 10,000 births [1] and is among the most frequent and severe developmental disorders of the foregut. EA results from incomplete separation of the ventral and dorsal parts of the foregut during embryonic development, leading to disrupted esophageal anatomy and often associated with tracheoesophageal fistula (TEF) [2]. After surgical repair of EA-TEF, children remain at risk for chronic respiratory and gastrointestinal morbidity, neurodevelopmental delay [3, 4] and impaired quality of life (QoL) [5, 6]. Children with long-gap EA (10–15%), managed by either delayed primary anastomosis or esophageal replacement [7], face increased risk for gastrointestinal disease [8], malnutrition [9], and pulmonary impairment [10]. Aerodigestive morbidity is especially pronounced in early childhood: 58% of young children with EA-TEF experience at least one chest infection per year [8], 54–61% have dysphagia [11], 65% have gastro-esophageal reflux disease [8] and 75% report feeding difficulties [12]. Furthermore, 55% of children with EA-TEF have associated anomalies, often requiring multiple medical and surgical interventions throughout childhood [7]. While international guidelines recommend lifelong multidisciplinary follow-up care for patients with EA-TEF [13], such care has not yet been consistently implemented in clinical practice [14].

Parents are the primary providers of their child’s physical, psychological, and social needs [15]. When children have chronic health conditions(CHCs), this role becomes even more central, encompassing the ongoing management of their child’s disease [16–18]. Parental involvement in managing the condition can directly influences the child’s health trajectory and developmental outcomes [15]. As a result, parents take on new roles and responsibilities balancing caregiving with family routines, work, and personal commitments, effectively extending their role as parent into that of a medical caregiver [17–20]. Furthermore, while families remain the primary source of support for school-aged children, early childhood is a critical period when condition management must expand beyond the home to include schools and the broader social environment [21, 22]. Parents may experience a high caregiver burden which is further compounded by challenges in navigating healthcare and educational systems to manage their child’s condition [18, 20, 23]. Parents of children with CHCs [24, 25], including those caring for children with EA-TEF, are at increased risk of elevated stress [26], mental health challenges [27, 28] and experience of family strain [29]. With EA-TEF, parents face challenges associated with their child’s aerodigestive symptoms [29], particularly feeding difficulties [29–31].

While parents’ experiences with condition management have been studied for many years within the broader context of “pediatric CHCs” [17, 18, 20], only has attention recently started to turn to the specific needs of children with rare diseases [32], including those with EA-TEF [30, 31, 33–36]. To gain a deeper understanding of children’s and parents’ experiences with EA-TEF, qualitative research has recently emerged in this field [30, 31, 33–35]. However, most patient- and parent-reported studies have originated from Europe [5, 6, 37] with limited contributions from the United States(US). To date, no study has specifically focused on parents’ experiences of condition management in EA-TEF. A deeper understanding of these experiences, particularly during early childhood, when morbidity is pronounced and the parent roles are being established, may help inform healthcare services and guide the development of targeted, family-centered supports for children with EA-TEF and their caregivers. This study therefore aimed to explore parents’ experiences with condition management in children born with EA-TEF during early childhood.

## Material and Methods

### Study design

**Fig. 1:**
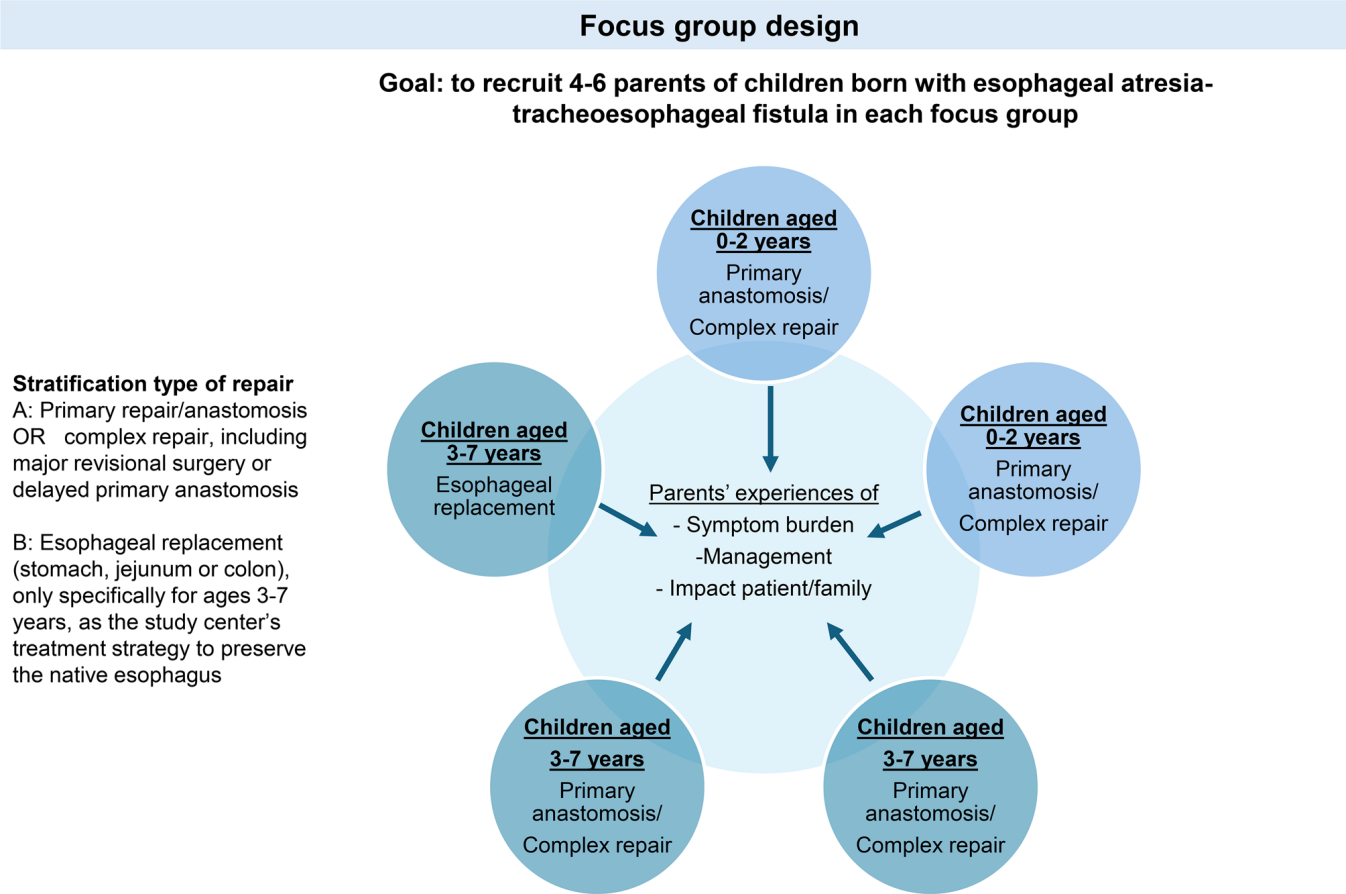
Illustration of study design to gain in-depth insight into parents’ experiences with condition management in young children with esophageal atresia-tracheoesophageal fistula

This research project forms part of a broader research initiative exploring patient and parent perspectives in EA-TEF (IRB-P00048407, IRB-P00646718). This specific study used a focus group (FG) design to gain in-depth insight into parents’ experiences with condition management in young children with EA-TEF, as illustrated in Fig. 1. Consistent with best practices for FGs, participant homogeneity was achieved by stratifying groups by child age and type of surgical repair. Five FGs were initially planned, with data saturation as the criterion for concluding data collection [38–40].

### Recruitment

**Fig. 2:**
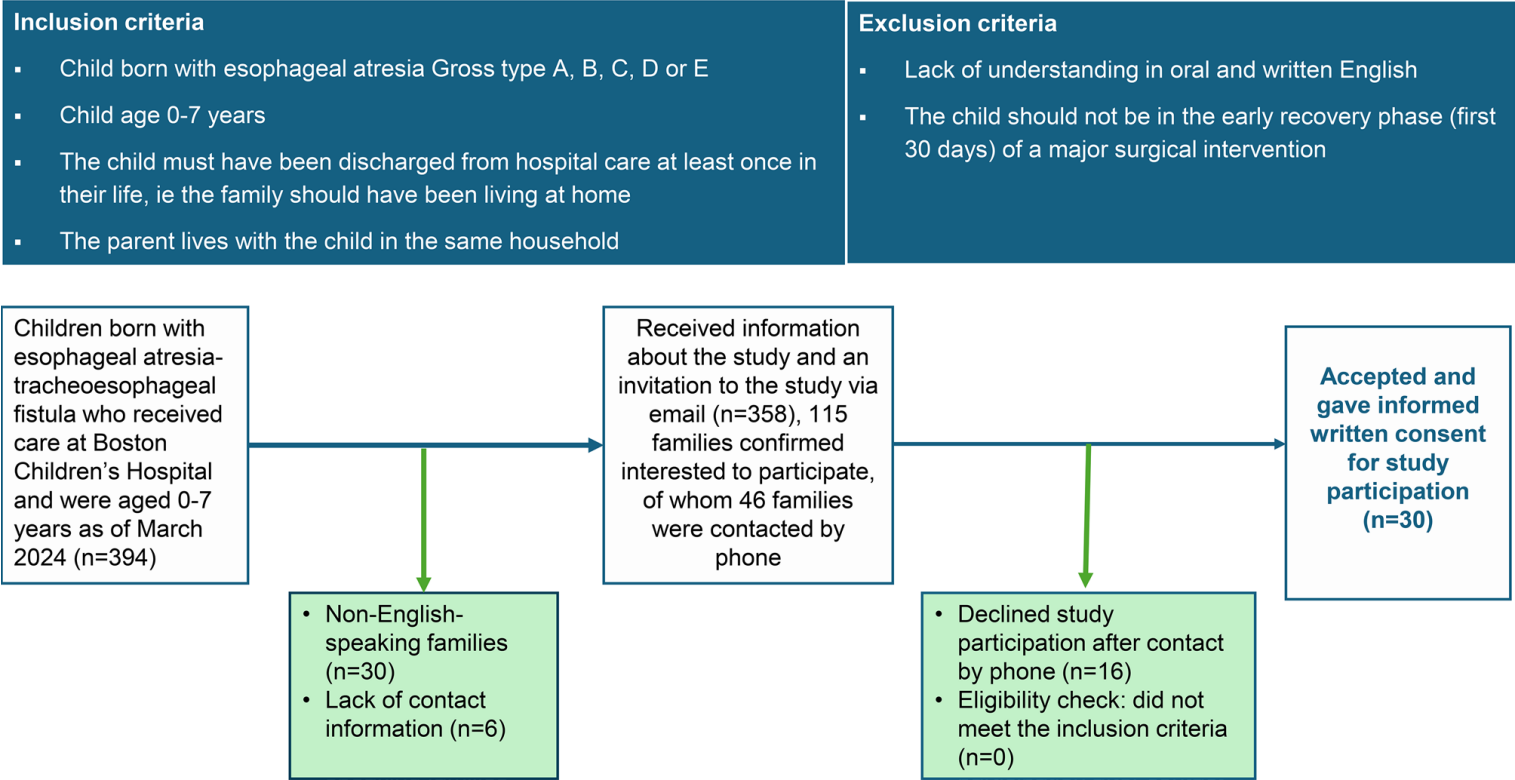
Flowchart of the recruitment procedure to five focus groups with parents of children born with esophageal atresia-tracheoesophageal fistula

Parents of children with EA-TEF who had received care at Boston Children’s Hospital (BCH), a tertiary pediatric surgical center specializing in multidisciplinary care of complex esophageal and airway conditions, were recruited purposively and conveniently, as detailed in Fig. 2.

### Data collection

Parents completed a sociodemographic questionnaire, and medical records were reviewed for neonatal and surgical history. FGs were conducted using a standardized semi-structured interview guide, developed based on prior content analysis of similar EA-TEF family FGs [41], input from a multidisciplinary research committee, and feedback from patient stakeholders in the EAT global support group. The guide covered three primary domains (Swallowing difficulties/Feeding/Dumping; Gastro-esophageal reflux disease; and Respiratory disease) with open-ended, probing sub-questions and follow-up questions exploring both child and parent’s experiences (Additional file 1). All FGs discussions were led by a trained moderator (MDB) who had previous experience in EA-TEF research and FG methodology, and no prior clinical relationship with the participants, ensuring that all participants had the opportunity to contribute. One field assistant (JB, LC, RM) was also present to document non-verbal cues and group interactions.

### Data analysis

The FG discussions were audio-recorded, transcribed verbatim and analyzed using manifest content analysis, a method for identifying patterns in text to group data into domains, typically referred to as categories. All subcategories and categories remained close to the participants’ original meaning, and no data was placed in more than one subcategory or category [42].

The content analysis started with one researcher (MDB) reading all FG transcripts. Meaning units relevant to the research question were extracted and merged with pseudonymized participant data in Microsoft Excel 365. The meaning units were condensed. Transcripts were read again by MDB alongside the meaning units to ensure all relevant content was captured. The condensed meaning units were then organized inductively and iteratively into codes, subcategories and categories. A final overarching thematic domain was subsequently developed to encompass the selected categories. The full categorization process was critically reviewed by five additional researchers (LC, RM, LF, KW, BZ), revisions were made collaboratively until consensus was reached among the authors.

Because FGs emphasize interactive discussion – allowing participants to explore experiences collectively and remain with topics meaningful to them [38, 39], quantifying each category helps illustrate the relative magnitude of reported experiences across the study population [42]. Participant statements within each category, along with study population characteristics, were analyzed using descriptive statistics (n, %) using Microsoft Excel 365. Representative quotes from different informants (I) were selected to illustrate how original parent statements aligned with the identified categories.

### Methodological rigor

As outlined in Additional file 2, methodological rigor was ensured in accordance with established principles of qualitative research [38, 43–45], and with Standards for Reporting Qualitative Research [46].

## Results

### Study population

**Fig. 3:**
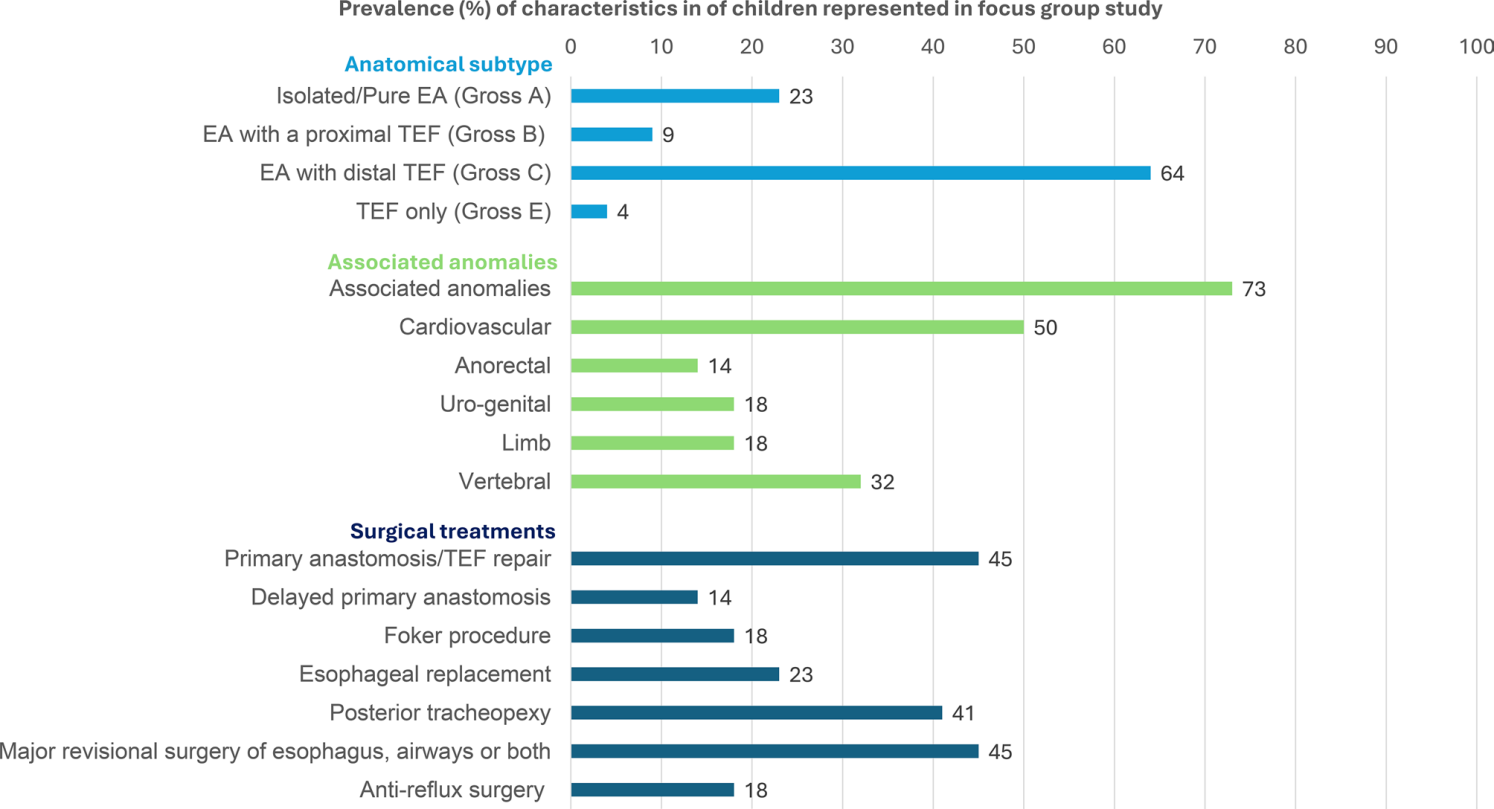
Presentation of characteristics of children represented in focus group study

Of the 30 parents who agreed to participate, 22 contributed to the FGs conducted in 2024, either in non-clinical settings at BCH (*n* = 4 FGs, primary anastomosis/complex repair) or via Zoom video meeting (*n* = 1 FG, esophageal replacement). Among the 22 participating parents (mean age 39; range 33–48 years), 82% were mothers, 94% had university/college education, and 9% were born outside the US. In nine families, there was a documentation of prenatal suspicion of EA in the medical records. The distribution of anatomical subtypes of EA-TEF, associated anomalies and surgical treatment (s) among their children (67% male) are presented in Fig. 3. At the time of the FG, 91% of the children were feeding orally, while 28% remained at least partially tube-fed via gastrostomy (23%) or jejunostomy (5%). Fourteen percent of the children had a tracheostomy and 55% a verified delay in neurodevelopmental function/milestones.

### Parents’ experiences

Parents’ experiences with condition management for children with EA-TEF during early childhood are organized into two overarching thematic areas. Table 1 gives an overview of categories and subcategories of the two thematic areas and are further described below. Data saturation was achieved across and within the FGs (Additional file 3).

**Table 1:** Overview of thematic area, categories, subcategories and illustrative quote on parents’ experiences of condition management in early childhood of children with esophageal atresia-tracheoesophageal fistula

	Categories	Subcategories	Quote
Thematic area 1: MANAGEMENT OF THE CHILD’S MORBIDITIES IN EVERYDAY FAMILY LIFE	Taking responsibility for the child’s health needs (213 statements, 22 parents)	• Managing respiratory symptoms and needs • Time, activities, and challenges around nutritional intake at home • Living and dealing with the child’s vomiting • Taking responsibility for medical treatment	“It affects every aspect of your life. Every meal becomes, you didn’t know you could concentrate this much on food” (I1)
	Challenges and possibilities in understanding the child’s condition/symptoms (90 statements, 21 parents)	• Challenges understanding the child’s symptoms • Communication with the child about their condition/symptoms • Noticing, seeing, and understanding the child’s symptoms	“I am happy that he’s older and can say stuff. ‘cause I do feel like I’ve been able to parent better and make better choices. “(I10)
Thematic area 2: NAVIGATING EXTERNAL SUPPORTS FOR THE CHILD AND FAMILY	Navigating through and finding supports in health care and community-based support programs (214 statements, 22 parents)	• Perceived insufficiencies in health care providers outside highly specialized care • Managing hospital visits and being the child’s advocate in the healthcare setting • The distance/closeness to specialized health care provider • Appreciation of a highly specialized health care provider • Needs and wishes to be provided by the health care provider • Use of community-based support	“I’m thankful for that relationship I created with our nurse practitioner because she kind of stuck with us. (I32)
	Managing the child’s transition into contexts outside home: Babysitters, daycare, and school (74 statements, 20 parents)	• Challenges in leaving their child in the care of others • Finding options and solutions for daycare/babysitting to work • Contact with the school and their level of understanding about EA-TEF • Difficulties and efforts around the child’s nutritional intake in school • Encountering challenges of school absence	”The feeding, or my child still has the G-tube in. I don’t know, we’re like, it’s not that hard, here’s how you do it. But it definitely limits the options in terms of the daycare” (I21)
	Defining the role of patient/peer support (38 statements, 10 parents)	• Needs and wishes for organized peer-support in EA-TEF • Use of existing parent-support	“We’re all on the same floor, wish for a quiet space for parents to talk to each other” (I22)
I, informant			

#### Theme 1: Management of the child’s morbidities in everyday family life

The first theme focuses on parents’ internal day-to-day responsibilities and included 303 statements from 22 parents.

**Taking responsibility for the child’s health needs**

##### Managing respiratory symptoms and needs (25 statements, 13 parents)

Parents described caring for their child’s respiratory infections, mucus issues, coughing and breathing difficulties. Parents consistently described respiratory infections as frequent and/or severe – referring to *“colds lasting forever”* (I14), being the *“biggest threat”* (I19), and symptoms that *“take over the house”* and *“interrupt everything”* (I11), disrupting both the child’s baseline health and family life. Parents focused on preventing respiratory infections and avoiding hospitalization. They developed family care plans, encouraged physical activity and physiotherapy, and took responsibility for closely monitoring the use of nebulizers and inhalers at home. To relieve mucous buildup, parents described strategies such as giving warm honey water to help loosen the secretions. Parents described efforts to reduce nighttime coughing and help their child sleep more comfortably. Some parents co-slept with their young child, keeping them in an upright position on their chest during episodes of breathing difficulty.

##### Time, activities, and challenges around nutritional intake at home (102 statements, 21 parents)

Parents who discussed their child’s feeding difficulties reported spending a significant amount of time and energy on feeding routines. Feeding was described as a *“full-time job”* (I1) or even a *“two person’s job”* (I13). Their role involved practicing feeding skills with the child and supporting their overall development. Parents described this process as a journey – marked by both celebrated milestones and frustrating setbacks in their child’s feeding progress. The start of oral feeding was described as a major milestone, particularly the moment when a child ate their first ice cream. *“Then, my child had her first tiny little scoop of ice cream and we have a video of it and we all celebrated ‘cause that was like a huge milestone”* (I18). Parents of children with a history of, or current reliance on enteral tube feeding described additional responsibilities - managing the tube, practicing oral feeds, and working to ensure their child maintained or gained weight. Many expressed both a desire and a struggle to wean their child off tube-feeding, when medically feasible. Parents made sustained efforts to encourage oral feeding – for example, using upright feeding positions, offering safe taste trials, gradually transitioning to solids, and learning which foods were tolerated versus those that caused stuckies. They also described taking steps to prevent stuckies, such as carefully measuring food portions, avoiding certain textures, adjusting mealtime routines, reminding the child to eat slowly, and following healthcare guidance. *“I have a stuckie radar”* (I2). Despite their efforts, parents still faced episodes where food became stuck, and their child gagged and/o coughed during meals. They also described feeding challenges, including food aversion after stuckies, selective eating behaviors, and a shared wish that their child had a stronger appetite or greater capacity to eat.

##### Living and dealing with the child’s vomiting (17 statements, 9 parents)

Parents described managing frequent episodes of vomiting - occurring during meals, episodes of crying or laughing, respiratory infections, or even as part of their child’s daily baseline. They recounted being vomited on during nighttime co-sleeping, or discovering vomit in the child’s bed the following morning. One parent remarked that *“My child’s puking was literally my life”* (I6). Parents of children prone to vomiting prepared by keeping buckets or napkins around at home for anticipated episodes. They elevated their child’s bed at night to reduce vomiting and promote better sleep. Additionally, parents observed that their child would intentionally induce vomiting to elicit a reaction or express distress. Parents expressed concern that persistent vomiting interfered with their child’s ability to gain weight and thrive.

##### Taking responsibility for medical treatment (69 statements, 18 parents)

Parents of children with complex EA-TEF described themselves as becoming, in effect, their child’s medical provider. Parents of children with tracheostomies described having to master equipment and procedures to routinely perform suctioning, reduce secretions, and clear the upper airway. One parent described managing a G-tube to provide emergency access in the event of low blood sugar. For respiratory issues, parents administered nebulizers and inhalers on routine schedule, especially *“when things ramp up”* (I12) and acted quickly to prevent severe infections and to *“calm the child’s sickness down”* (I12). Some parents initiated antibiotics preemptively to prevent pneumonia from developing. Parents also reported difficulties with getting their child to take medications, especially antireflux medication. They monitored medication supply closely and were prepared to stockpile medications to avoid running out and ensure continuous treatment. They also described challenges in deciding whether a medication was effective and when it was appropriate to wean their child off it. *“Just deciding if my child should be on the antireflux medication, am I making the right choice? […]it still scares me. Should I have kept my child on it?”* (I3)

**Challenges and possibilities in understanding the child’s condition/symptoms**

##### Challenges understanding the child’s symptoms (31 statements, 13 parents)

Parents described that their child’s limited ability to communicate symptoms, due to their young age or cognitive challenges, made it difficult to interpret issues such as vomiting, food getting stuck, heartburn, reflux, cough, aspiration, or feeding difficulties. These challenges lessened as children developed the ability to verbalize their sensations. Before their children could communicate verbally, parents relied on and trusted practitioner’s physical assessment for clinical insight. *“My child can have a stuckie in, you wouldn’t even be able to tell”* (I11). Parents found it difficult to determine whether symptoms were related to EA-TEF or not, especially when evaluating symptoms against an already poor baseline. *“Hearing my child sounded like a bulldog and wondering, is he ok?”* (I17). Parents also noted the challenge of interpreting symptoms when there were no visible signs – *“You couldn’t always see on the outside of the child that anything is wrong”* (I6). Parents expressed uncertainty, sometimes not knowing or only suspecting symptoms such as heartburn, reflux, feeding difficulties or mucus problems. They also struggled to understand how their child’s symptoms were interrelated, or even how to label or describe them accurately.

##### Communication with the child about their condition/symptoms (15 statements, 4 parents)

Parents of children aged 5 years or more reported feeling more confident in understanding their child’s condition when they were able to communicate symptoms such as heartburn, pain, food getting stuck or mucus problems. Parents also described receiving questions from the child about their symptoms, the reasons behind them, their EA-TEF diagnosis, and need for hospital visits. They tried to answer and discuss these EA-TEF-related questions openly with their child. When hospitalization was mentioned, some children reacted emotionally - crying or showing distress - and parents provided reassurance and comfort.

##### Noticing, seeing and understanding the child’s symptoms (44 statements, 15 parents)

Parents described learning to observe their child’s symptoms closely – particularly during meals and physical activity. They paid attention to behavioral cues – such as lack of appetite, gagging, gurgling or facial expressions when experiencing a stuckie), including how to identify when symptoms were at their worst or to recognize the child’s *“biggest hurdle”* (I22). Parents expressed developing a strong intuition, *“a gut feeling”* (1), about their child: recognizing when something was wrong, which medications were effective, how their child responded to illness e.g. respiratory infection, desaturation, suctioning needs, distress and what they needed. Over time, they reported becoming familiar with their child’s baseline, such as their typical cough. They learned to interpret respiratory sounds, such as “cracky” or “yucky” chest sound, the depth of secretions, or a brewing noise. They also observed the amount/degree of symptoms, such as large amounts of mucus the child could expel and made inferences about potential underlying causes. Furthermore, parents observed that their children also understood their own symptoms, such as aspiration, heartburn, reflux, or vomiting, and were able to make their own observations. In these cases, parents expressed confidence in understanding and managing their child’s symptoms.

#### Theme 2: Navigating external supports for the child and family

The second theme addresses how parents engage with healthcare and community systems to support their child’s health and development and included 326 statements, 22 parents.

**Navigating through and finding supports in health care and community-based support programs**

##### Perceived insufficiencies in health care providers outside highly specialized care (65 statements, 19 parents)

Parents described experiences of shortcomings among healthcare providers outside highly specialized centers, including delays in diagnosis and EA repair, misinterpretation of symptoms, and complications they attributed to a lack of EA-TEF knowledge among their initial healthcare provider(s). *“It has been hell on earth for us, for the first three years of my child’s life, I think we hold on to quite a lot of resentment towards our initial care team that, quite frankly, reached the end of their runway, if you will, as far as their own skills”* (I21). Parents also described a lack of recognition for their role, inadequate listening by providers, not being taken seriously, and limited involvement in decisions about their child’s care. They reported receiving insufficient information about surgical risks and described feeling misinformed by providers. Parents expressed distrust of these health care providers. They also noted poor collaboration between medical specialties and departments, which they felt compromised the quality of care their child received. Parents described navigating fragmented health systems often reaching specialized care only after experiencing *“botched surgeries”* (I9), other complications or by sheer luck of living near a specialized healthcare provider.

##### Managing hospital visits and being the child’s advocate in the healthcare setting (57 statements, 19 parents)

Parents reported frequent hospital visits and admissions related to health monitoring, treatment, and the need for surgery. They also described seeking emergency care when their child’s respiratory infection worsened, sometimes resulting in intensive care unit admissions. Wanting their child *“to feel normal”* (I6) and like *“a complete person”* (I6), parents explained how they took on the role of advocate during every medical consultation and care encounter. They advocated for key care decisions, such as when and how to wean from tube feeds. Parents often found themselves educating non-specialized healthcare providers on how to manage their child’s EA-TEF. They described repeatedly having to explain their child’s needs and occasionally needing to *“make a stink”* (I4) when something did not feel right. Parents also described the need to coordinate between highly specialized care centers and local providers to ensure their child received consistent, high-quality care. They researched the child’s condition, came prepared to ask, *“a billion-and-a-half questions”* (I4) and sought out the best methods and providers, especially for surgical procedures, to ensure their child received appropriate care and support.

##### The distance/closeness to specialized health care provider (10 statements, 5 parents)

Parents noted that accessing highly specialized care for EA-TEF was often difficult. Those who were able to access it expressed gratitude. The expertise of specific doctors was so highly valued that parents followed them across the country and, in some cases, even based their family’s location on proximity to that provider. Some parents were willing to relocate, if necessary, while others were unable to do so and found the long distance to a trusted health care provider challenging.

The remaining three subcategories “**Appreciation of a highly specialized health care provider”, “Needs and wishes of the health care provider**” and “**Use of community-based support**” are summarized in Table 2. As shown, parents expressed appreciation for coordinated and available care teams that were responsive to the child’s and family’s needs. This was also reflected in their identified needs and wishes from their health care provider. In addition, parents described the need for clear referral pathways to the appropriate provider and strong coordination between the specialized and local provider. Parents also said accessing community-based support was helpful to support their child’s development.

**Table 2:** Presentation of the subcategories “Appreciation of highly specialized health care provider” and “needs and wishes of their health care provider” identified by parents of children with esophageal atresia-tracheoesophageal fistula, as described during focus groups

Subcategory	Quote
**Appreciation of a highly specialized health care provider (50 statements, 17 parents)**	
• Appreciation for care teams that prioritized their child’s well-being, and responded sensitively to the needs of both the child and family • Importance of a coordinated multidisciplinary team, one that collaborated effectively, involved parents in decisions, listened to their input, kept them well informed, and maintained open communication • Importance of: ◦ Feeling “on the same page” (I4) with providers ◦ Timely access to care ◦ Proactive approaches by the care team ◦ High level of clinical expertise among providers, which gave them ▪ peace in mind ▪ a sense of security ▪ gratitude that their child could receive appropriate care ◦ Strong collaboration between specialized and local health care teams, allowing ▪ expert recommendations to be communicated and implemented at the local level • Timely answers to their questions and follow-up on their concerns	“They’ve [health care provider] been very thoughtful about supporting us and what we wanna do, but at the same time, really proactive” (I17)
**Needs and wishes from health care providers (24 statements, 11 parents)**	
• Focus on the child’s best interest • Collaboration between providers and parents • Listening to parents and taking their concerns seriously • Opportunities for parents to ask questions and receive guidance • Access to integrated multidisciplinary care • Provision of post-operative follow-up care • Strong coordination between specialized and local providers • Clear referral pathways to the appropriate provider • Respect for parents’ experiential expertise	“We become parents to disabled children, we become medical providers without a degree, and there’s no better experience than hands-on. So I think that’s something that they need to really listen” (I16)
**Use of community-based support (8 statements, 4 parents)**	“During that time, the support of home and community-based services has been really important” (I21)
• Accessing helpful, family-centered community-based services that supported their child’s development, including feeding	

**Managing the child’s transition into contexts outside the home: babysitters, daycare, and school**

This category includes a description of challenges, options and solutions parents experienced during their EA-TEF child’s transition into contexts outside home and is detailed in is detailed in Table 3. Parents identified both external challenges, such as difficulties finding a babysitter, as well as internal personal difficulties, such as the desire to stay in control, fear or distrust to others, that hindered them leaving their child in the care of others. At the same time, parents described solutions for daycare/babysitting. In contact with school, they faced lack of understanding of EA-TEF and faced concerns from teachers regarding their child’s cough or vomiting. Specific difficulties were centered around the child’s nutritional intake in school. Parents also expressed encountering challenges of the child’s school absence due to EA-TEF.

**Table 3:** Description of subcategories of the category “managing the child’s transition into contexts outside the home: babysitters, daycare, and school” with the number of statements and parents adding to the subcategory

Subcategory	Quote
**Challenges in leaving their child in the care of others (18 statements, 12 parents)**	
• Difficulty finding a babysitter due to others feeling scared, nervous or even terrified to care for their child • Lack of an extended family who understood the situation or were capable to providing care • Desire to stay in control, fearing that something might happen to their child in their absence • Distrust in allowing others to handle their child’s oral feeding, contributing to hesitancy around starting daycare	“You don’t want to give up that control because you’d rather, if something went wrong or something happened, it happened on your part” (I1)
**Finding options and solutions for daycare/babysitting to work (17 statements, 9 parents)**	
• Relief when they found a trustworthy babysitter or daycare solution, making it possible to leave their child in someone else’s care • Need to remain available to answer ongoing questions from babysitters or daycare staff about their child’s feeding • Support from assigned nurse who, in some cases, served as the child’s babysitter or accompanied them to daycare/school • Active search for daycare solutions willing to accept their child, even if the child was tube fed • Appreciation for the ability to work from home, allowing their child to delay entering daycare or preschool	“Luckily, like find a great daycare that’s been really willing to work with us through his various feeding” (I15)
**Contact with the school and their level of understanding about EA-TEF (13 statements, 7 parents)**	
Need to repeatedly explain their child’s condition, particularly baseline EA-TEF symptoms, to school staff and teachers Lack of understanding about EA-TEF among school staff Concern from teachers when child coughed or vomited, often requesting in parent contact and requests for the child stay home Lack of awareness of EA-TEF in school as frequent source of frustration. Mixed experiences with school support, with some describing ongoing struggles to gain teachers understanding and others felt trusted and supported by school staff	“School [staff] are always concerned he’s coughing and should stay home” (I11)
**Difficulties and efforts around the child’s nutritional intake in school (16 statements, 5 parents)**	
• The child’s nutritional intake at school as a major concern • While they recognized the importance of their child sitting and eating with peers, parents expressed discomfort with leaving oral feeding responsibilities to school staff • One option for parents was to not allow their child to eat in the lunchroom at school • Another option was to prepare home lunch with safe foods for their child to bring to school • In some cases, parents successfully arranged for a teacher or nurse to sit with their child during lunch	“We really felt strongly that maybe the lunchroom wasn’t the best place for my child for a little while” (i18)
**Encountering challenges of school absence (10 statements, 3 parents)**	
• Child sent home from daycare/school due to baseline EA/TEF symptoms • Frequent or prolonged absences due to respiratory infections, medical treatments and hospital visits • Difficulty deciding whether to send their child to school when the child had a cough or was recovering from hospitalization • Contact from school staff regarding their child’s frequent absences	“And so I got a letter from the school saying they reported us […]. ‘Cause my child had more than 19 absences.” (I9)

EA-TEF, esophageal atresia-tracheoesophageal fistula

**Defining the role of patient/peer support**

The category “Defining the role of patient/peer support” was built up by the two subcategories **“Needs and wishes for organized peer-support in EA-TEF”** and **“Use of existing parent-support”** and are explained in Table 4. As described, parents expressed a desire to connect with other families of children with EA-TEF, noted the lack of organized peer support groups, but recognized that the timing and need for peer support may vary across families. They also declared pros and cons by using existing parent-support via social media.

**Table 4:** Description of subcategories of the category “Defining the role of patient/peer-support” with the number of statements and parents adding to the subcategory

**Needs and wishes for organized peer-support in EA-TEF (15 statements, 6 parents)**	**Quote**
• Strong desire to connect with other families of children with EA-TEF, to know “that you are not alone” (I2), “that somebody knew what that felt like” (I14) and “to have someone [who] really truly knows what you’re going through” (I16) • Recognition of the neonatal intensive care unit as a potential important setting for early connection among families • Noted lack of organized peer support groups or parent meetings, with interest in flexible opportunities to participate when ready • Acknowledgment that the timing and need for peer-support may vary across families • Value in listening to others who had been through similar experiences, seeing pictures of children eating and learning from success stories	“And for my child, in the future, maybe to have peers that have […] So my child can bounce off of them and be like, Hey, do you throw up when you eat, too?” (I14)
**Use of existing parent-support (23 statements, 6 parents)**	
• Listening to and sharing experiences with other parents • Using social media parent groups (e.g. Facebook) to connect, share knowledge and receive support from other families • Challenges of social media groups such as exposure to worst-case scenarios • Benefits of social media groups: ◦ Connecting with other parents even before their child was born ◦ Receiving recommendations for experienced EA-TEF providers ◦ Learning about effective medications, and preparing informed questions for medical appointments	“I’m just seeing pictures of kids eating when I was going through it, I was like, okay, he’s gonna be able to eat. It is super helpful” (I16)

EA-TEF, esophageal atresia-tracheoesophageal fistula

## Discussion

The FG study demonstrated that, within the U.S. context, parents of children with EA-TEF experienced condition management during the first years of their children’s lives as a significant responsibility, having an ongoing caregiving role to address their child’s health-related needs. This includes the day-to-day oversight of respiratory, nutritional, and medical care at home, the interpretation and response to symptoms, the coordination of healthcare services, advocacy within medical and educational settings, and efforts to ensure safe and developmentally appropriate participation in broader social environments such as daycare and school.

While condition management from the perspective of parents of children with CHCs has long been studied [17, 18, 20], EA-TEF remained absent from this body of research. As the first reported FG study on this topic, our results suggest that caregiving a young child with EA-TEF includes both condition-specific demands and responsibilities common to CHC. In particular, management of their child’s co-morbid respiratory disease, feeding and recurrent vomiting problems emerged as central features that appear especially characteristic to EA-TEF. Parents in this study recognized their child’s feeding as a major responsibility and area of focus. Feeding difficulties in children with EA-TEF are related to parental experiences of trauma, anxiety, worry, fear and uncertainty [28, 30, 31]. However, less is known of parental management of their child’s respiratory morbidities. Our study further highlight the central role of respiratory morbidity in shaping parents’ caregiving experiences. Parent described constant vigilance to their child’s respiratory health, and the ongoing “threat” or actual occurrence of frequent and severe respiratory infections to have a significant impact on both the child and the family. Severe tracheo-bronchomalacia in children with EA-TEF, which is associated with such respiratory infections [47], has previously been identified as an independent predictor of high family burden in children with EA-TEF [29]. The fact that 41% of the children in our study were treated with tracheopexy address severe tracheo-bronchomalacia, which may help to explain these parents’ experiences. Furthermore, parents were considerably affected by their child’s vomiting problems, which has earlier been shown to be associated with impaired QoL of children with EA-TEF [48]. In our study, parents’ experiences demonstrated heightened vigilance to their child’s respiratory, feeding and vomiting problems, including careful monitoring for possible deterioration and need for medical treatments. This vigilance has also been observed in other CHCs, with parents stating that they remain constantly on guard to intervene promptly to protect their child [16–18, 49]. Parents of children with complex EA-TEF morbidities expressed to often take on the role of medical providers for their child. Such responsibility of condition management can be onerous for parents [16, 49]. Parental responsibilities of caring for young children with EA-TEF in everyday life conceptually agree with that described in other CHCs, such as the experience of significant responsibility, heightened vigilance and the feeling of being a medical provider [20, 21], but is in its context closely tied to the child’s aerodigestive function.

This study also illustrated how parents of children with EA-TEF make efforts to master understanding of their child’s symptoms/condition during early childhood. While challenges to achieve such understanding were described, it is noteworthy that uncertainty around the child’s condition can be stressful for them [16]. We found that parents children with cognitive dysfunction expressed challenges to understand their child’s symptoms/condition. In our study 55% of the children presented with a verified delay in neurodevelopment according to medical record review. Unfortunately, such prevalence is difficult to compare in the EA-TEF literature, as neurodevelopment has been differently assessed, with contrasting results [3]. However, a recent national population-based registry study demonstrated that individuals with EA-TEF have a higher risk of intellectual disability than general controls [50]. While parents’ of the youngest children also expressed challenges to understand their child’s symptoms/condition, other studies have shown that parents of children with CHCs learn from each illness episode and daily caregiving, using their experiences to identify and respond to illness symptoms in their child [20]. Similarly, parents in our study developed an intuition and knowledge of their child’s baseline condition, which may help increase their confidence as primary caregiver [49]. Interestingly, parents in this study began discussing EA-TEF with their child at 5 years of age. This is important as it reinforces the child’s understanding of their condition and supports their QoL [41, 51].

In this study, the majority of parents experienced insufficiencies with outside specialized healthcare, which they believed negatively impacted their trust and adversely affected their child’s health outcomes. Caregiver burden can stem from inadequate support systems, resulting in parental isolation in their caregiver role and barriers to high quality care for their child [20, 23]. If a health care team is not forthcoming, the parents need to balance assertiveness against the risk of alienation of those they need for support in their caregiving role [49]. Parents perceptions of deficiencies outside highly specialized care such as not being taken seriously or listened to, lack of information, lack of involvement in decision making of the child’s care are consistent with reports from parents of children with other CHCs [18]. Perhaps in response to such experiences, parents in our study assumed the role of advocate for their child. Such advocacy has been observed in CHCs [23, 52] and included parents need to speak up, ask questions to their provider [52] and to stand up for their needs [23], but has remained underexplored in EA-TEF and represent a critical component of condition-management in this population.

At the same time, parents in our study expressed appreciation for highly specialized care teams, and identified needs/wishes of their health care provider. They valued similar qualities of their health care providers as found in other CHCs; mutual trust between parent-provider, listening, partnership, clear and honest communication, compassionate care, recognition as the child’s expert and meeting a provider who knows their child and the family [23, 52, 53]. Interestingly, these priorities agree with principles of family centered care [54], yet have not been recognized in previous studies of EA-TEF. Parents’ preferences for access to a multidisciplinary team with high competence in EA-TEF, aligns with a model of centralized care for rare diseases [55], which aim to concentrate clinical experience and improve care. Of note, these aspects were sometimes so important to families that they were prepared to relocate to remain close to the appropriate health care provider.

Importantly, parents’ experiences in this study cannot be understood independently to a broader healthcare context, but can instead illustrate how community- and societal-level factors influence parental responses to health care services [22]. Within the U.S. healthcare and insurance-based system, access to specialized pediatric centers is shaped not only by a child’s clinical need, but also by insurance coverage, financial resources, and geographic proximity. Parents’ experiences of challenges related to fragmented healthcare systems have been reported in other countries like United Kingdom and Australia, as contributing to perceived gaps in support and to parental struggles to obtain adequate services [56, 57]. Together these findings conceptually reflect a transfer of responsibility for the child from healthcare systems to families, accompanied by increased reliance on alternative sources of support, such as charities and peer support groups [58]. Additionally, in this and another study [59], the U.S. insurance-based system can be understood as creating disparities in who is able to access and sustain care at expert institutions, and increasing pressures for family relocation. In line with this, Validova et al. [60] recently showed that underinsurance in the U.S. system is associated with increased severity and complexity of pediatric conditions.

We found that parents’ condition management included supporting their child’s transition into social settings (e.g., babysitters, daycare and school), and such transitions presented challenges, especially related to feeding. Swedish studies have shown that 20% of children with primary anastomosis and 60% of children with long-gap EA utilized school-based nutritional support [61, 62]. In this study, school absence was also raised as an issue among the subgroup of families whose children reached the age of starting school. In comparison, 29–36% of children with EA-TEF have previously been reported to have ≥12 school absences a year. Such high school absence also independently predicts worse family functioning in children with EA-TEF [29], and is associated with their reduced school functioning and use of special school support [61].

Moreover, this study identified patient-peer support as a possible external resource for parents of children with EA-TEF. De Vos et al. recently showed the beneficial impact of tailored EA-TEF support groups on parents’ emotional well-being, benefiting both those who share personal experiences and those who receive support [36]. Parents of children with EA-TEF may also turn to a support group to gain reliable information [31], which is similar to findings in other CHCs [18].

### Study strengths and limitations

This study addresses an underexplored aspect of EA-TEF and focuses on the concept of “condition management” from the perspectives of parents of young children, as part of a broader research program. This FG study included a greater number of participants than many previous qualitative studies on the topic [33–35], with data saturation achieved [38]. Methodological rigor was ensured throughout the study [43, 44]. Still, the generalizability of FG findings is limited [38, 39]. Findings should be interpreted within the context of the study setting. Although the geographical uptake within US was enhanced by offering a virtual FG and stratification for surgical type of repair was used to improve representativity in EA-TEF, BCH is a highly specialized center for esophageal and airway surgery, including complex revisional surgeries after failed repairs performed elsewhere. Additionally, the structure of the U.S. health care system differs substantially from that of other countries, potentially limiting transferability. The parents in our study had access to highly specialized care for their children, which may not reflect the experience of many other families. They were also mostly highly educated mothers; however, data on educational level from eight parents were missing. The overrepresentation of mothers has been noted in other studies and attributed to the high rate of mothers acting as primary caregivers [20]. Socioeconomic factors, including parental unemployment, lower educational attainment and less health insurance coverage are known determinants of caregiver burden and poorer child health.-related QoL [63–65]. Within the US healthcare and insurance-based system, unequal access to expert institutions, may further exacerbate these disparities, potentially amplifying parental burden and adverse child outcomes. As a result, the perspectives captured in this study may underrepresent families facing the greatest structural barriers to care and this limits transferability of our findings. Moreover, the quality FG data is highly reliant of the moderator skills. In this study, a trained moderator with no clinical relationship with the study participants used a semi-structured interview guide, to enhance consistency across groups and achieve neutrality [39].

### Implications for clinical practice and research

In our study, parents of nine children were informed of suspicion of EA-TEF before birth of their child. However, being informed about the diagnosis EA-TEF either pre- or postnatally is associated with increased stress levels in parents [66] and may lead to emotional turmoil [16, 23, 34, 53]. In EA-TEF, 60% of parents reach levels of post-traumatic stress syndrome [26]. This suggests that parents of young children with EA-TEF often carry significant responsibilities in managing their child’s condition while simultaneously facing emotional vulnerability. Although several parental responsibilities of caring for young children with EA-TEFs conceptually overlap with those reported in other childhood CHCs [20, 21], identifying these responsibilities within a condition-specific context is crucial. For families affected by EA-TEF, challenges are closely linked to their child’s aerodigestive function, which is critical for survival and development. Parent described witnessing respiratory distress, cyanotic episodes (“blue spells”), prolonged respiratory infections, choking events, frequent vomiting, and ongoing difficulties with weight gain and thriving [30, 67]. These events are stressful for parents [29, 30], while they also represent essential clinical information that parents must be able to communicate effectively to healthcare providers. In this study, a central driver of parents’ everyday condition management was the need to secure appropriate services that aligned with their child’s evolving needs. This finding highlights the importance of healthcare providers listening attentively to parents’ perspectives of their child’s aerodigestive function and valuing their experiential knowledge as a critical component of clinical decision-making [23].

Given that external systems, including healthcare providers and educational services, affect individual and family functioning [21], our findings highlight the need for healthcare systems that prioritize centralization of EA-TEF expertise and coordinated multidisciplinary care. Such teams should include, at a minimum, a pediatric surgeon, specialist nurse, gastroenterologist, pulmonologist, dietician, speech therapist, psychologist and social worker. These professionals can provide anticipatory guidance at key developmental milestones, including before the first hospital discharge and during transitions to daycare and school. This may include a) written information of EA-TEF b) predefined feeding and respiratory safety plans and c) structured communication between healthcare and schools. Such multidisciplinary teams could take responsibility for coordination of care plans of children with EA-TEF across health care providers that have included parents in care/treatment decisions. Within multidisciplinary teams, designated roles, such as a specialist nurse, psychologist and/or social worker may be particularly important in addressing parents’ psychosocial needs and supporting their parental role. Coordinated care models that actively include parents in treatment planning and decision-making may help reduce caregiver burden and strengthen parent-provider relationships. Despite the recognized importance of family-centered approaches, a recent systematic review [68], highlights an alarming absence of research on family-centered interventions for children with congenital malformations, with existing studies limited to children with congenital heart disease. This highlights an urgent need for future research to develop and evaluate such interventions tailored to EA-TEF, including the role of peer-support programs and their impact upon child and family outcomes. Moreover, the needs of both parents and children should be integrated into care guidelines for EA-TEF, such as those established by the International Network of EA [13].

## Conclusion

Within an insurance- driven healthcare system, parents of children with EA-TEF carry a significant and ongoing responsibility in managing their child’s aerodigestive disease, and are vigilant in trying to respond to their needs to protect them from complications. To ensure their children receive adequate support, parents often become advocates in the healthcare setting, navigating challenges across healthcare, educational services and patient/peer support systems. Their experiences point to the pressing need for compassionate, coordinated, multidisciplinary care models that actively partner with families, reflecting core principles of family-centered care. To improve outcomes for children with EA-TEF and their families, clinical practice and healthcare delivery must deliberately integrate parents’ voices and lived experiences into care planning and decision-making.

## Electronic supplementary material

Below is the link to the electronic supplementary material.

Supplementary Material 1

Supplementary Material 2

Supplementary Material 3

Supplementary Material 4

## Data Availability

The datasets analyzed during the current study are available in the manuscript or in its additonal files. Further information is not available to the public due to lack of ethical approval.

## Abbreviations

EA: Esophageal Atresia
FG: Focus Group
TEF: Tracheo-esophageal fistula
TBM: Tracheobronchomalacia
QoL: Quality of Life
US: United States
