# Supplementary material for: Parents’ experiences of condition management in children born with esophageal atresia-tracheoesophageal fistula during their early childhood
Source: Orphanet J Rare Dis. 2026 Feb 27;21:122. doi: 10.1186/s13023-026-04288-4 (PMC13040731; doi:10.1186/s13023-026-04288-4)
Supplement: Supplementary file 1 — Supplementary Material 1 [file 13023_2026_4288_MOESM1_ESM.docx]

The interview manual presented below is part of a standardized focus group manual developed to explore disease-specific experiences in everyday life of children and adults born with esophageal atresia and pre-testing a set of symptom burden and treatment experience measures. To increase its patient-centeredness, the interview manual build upon domains that have been identified following content analysis of focus groups conducted with families of children with EA at an earlier stage. The interview manual will focus on questions that facilitate exploration and the participants’ stories and experiences in their everyday life and try to ensure that these main domains are fully explored from the patient and parent point of view. The questions will be standardized and therefore divided into the following domains; 1) Swallowing difficulties, Mealtime adjustement, Selective/Restrictive Eating, Dumping; 2) Gastro-esophageal reflux disease; 3) Respiratory Disease.

##

## Core questions to parents of children 0-7 years

When I [the moderator] will use the term esophageal atresia, I wonder if this is the word you usually use to describe your child’s health condition or which term you use? The moderator will make sure that the participants understand and feel comfortable with the use of the term esophageal atresia, and if needed, will adjust the term.

| *The moderator will bring these questions to the focus group and check the box when the questions and domains have been discussed by the study participants. The field assistant will make field notes according to each domain. The sub-questions should be asked if those aspects are not already embedded and covered in the parents’ narratives. Probes only used as necessary. Pre-defined to standardize the manual.* | |
| --- | --- |
| Domain 1. Swallowing difficulties, Mealtime adjustment, Selective/Restrictive Eating, Dumping |  |
| *Moderator: Think about your child’s eating. With eating we mean breastfeeding/bottle feeding/tube feeding and/or eating food by mouth. We would like you to describe how does eating work out for your child.*  *First you will be able to discuss aspects for children who have started eating food by mouth, then for children who are solely tube fed and last, we will focus on potential symptoms your children experience during their feeding situation.* |  |
| 1. Have your children started eating food by mouth? If so,  - How does a typical feeding situation for your child look like? - How did you experience their transitioning from tube feeds to oral feeds? - Which experience do you have of your child’s processing food in their mouth? Probe. Do they need to chew carefully, store or spit out food? Please explain to me how it works. - What size of a meal can your children eat compared to their peers? - Which foods are difficult or easy to eat for your children? Compared with peers/siblings? - Do they have special modifications to feeding by mouth (Thickening, different type of bottles/formula/nipples/straws, etc)? - How much time do your children need to finish a main meal? Compared with peers? - How would you describe your children’s appetite? Probe. Hunger, feeling of fullness? - Which foods does your children enjoy or do not enjoy eating? Probe. Please give me examples. - What experiences do you have of your child’s feeding outside home? Probe. Nursery/Preschool?  1. If your child does not eat by mouth, can you describe the barriers for doing so? (Probe: oral aversion, aspiration, lack of interest, difficulty eating?)  - How do you tube feed your child? Please describe a typical tube feeding situation for your child. - How do you children experience, behave or react during the tube feeding situation? - In what way has tube feeding impacted your child’s health? Probe: physically, socially, psychologically? - In what way has tube feeding impacted your situation as a parent and family?  1. Think about your child’s eating again. How does your child feel and function while eating, do they experience food getting stuck, coughing, choking, vomiting? If so, please describe a situation, when your child experienced any of these symptoms?  - When does this happen? Please give some examples. - How often does your child experience…? - How has it been for your child to…? - What does your child do when they experience these symptoms? - What happens after your child has eaten? How do they feel? |  |
| Given what you have told me here today, what troubles in eating situations for your child are most important for us to know about? |  |
| Is there anything we have forgot to discuss in relation to your child’s eating, before we move on to the next session? |  |
| Domain 2. Gastro-esophageal reflux disease |  |
| *Moderator: Some children with EA encounter symptoms related to reflux like heartburn, vomiting, sensation of foods coming up or burps, chest pain. I would like you to tell me stories so that I can understand which symptoms does your child experience.* |  |
| Which symptoms of reflux have your child experienced during the day or night? Please describe a situation, when your child experienced any of these symptoms?   - When does this happen? Please give some examples. - How does your child describe that this symptom feels for them…? - How often does your child experience…? - How has it been for your child to…? - What does your child do when they experience these symptoms? |  |
| Given what you told us, what symptoms of reflux are the most important for us to know about? |  |
| Is there anything we have forgot to discuss about reflux, before we move on to the next session? |  |
| Domain 3. Respiratory Disease |  |
| *Moderator: Some children with EA may also experience symptoms related to their respiratory function. I would like you to tell me stories so that I can understand which symptoms your child experience.* |  |
| Which experiences does your child have regarding cough? |  |
| Which experience do you have regarding your child’s mucus problems either during the day or night? |  |
| What does your child do when they experience these symptoms? Which experience do you have regarding your child’s feeling of tightness in their chest? |  |
| Please tell me about your child’s physical strength, your child’s energy and how they keep up during physical activity such as play. |  |
| Which experience does your child have regarding shortness of breath? |  |
| Which experience do you have regarding respiratory infections? |  |
| For every subdomain, ask participants to tell me more… Probe. Please describe a situation, when you experienced any of these symptoms.   - When does it happen? Please give some examples. - How often does your child experience…? - How does this symptom feel for your child…? - How has it been for your child to…? - What does your child do when they experience these symptoms? - What makes it better or worse? |  |
| Given your descriptions/accounts here today, what respiratory symptoms are most important for us to know about? |  |
| Is there anything we have forgot to discuss before we move on to the next session? |  |

## Examples of follow-up questions used by the moderator

- In your view, what is the worst symptom for your child while….? Probe. Please tell me about a situation when your child experienced this symptom.
- In your view, what troubles during…. have been least bothersome for your child? Probe. Please tell me about a situation when your child experienced this symptom.
- How have these symptoms or troubles changed after your child’s treatment? Probe. What has been helpful and unhelpful for you and your child?
- Can you describe a situation where you experienced…?
- How has it been for you as a parent?
- How did that happen?
- How did this affect you?
- What does that mean for you..?
- How often do you experience…?
- When do you experience…?
- What has been worst…?
- What was most difficult…?
- What was the least bothersome…?

## Examples of questions that help create group discussion

Return the question to the group members with body language and words

- “If you all compare with your experiences, do you agree or disagree/think same or different?”
- What does everyone else think?
- Do others have different thoughts? Tell me more…
- When you hear this, what is your experience?

## Examples of how to pick up the non-verbal language in the group

- I can see you are nodding/shake your head. What were you thinking?
