# Supplementary material for: Parents’ experiences of condition management in children born with esophageal atresia-tracheoesophageal fistula during their early childhood
Source: Orphanet J Rare Dis. 2026 Feb 27;21:122. doi: 10.1186/s13023-026-04288-4 (PMC13040731; doi:10.1186/s13023-026-04288-4)
Supplement: Supplementary file 3 — Supplementary Material 3 [file 13023_2026_4288_MOESM3_ESM.docx]

| Additional file 2A. Presentation of concepts and their consideration in the present study to ensure methodological rigor | | |
| --- | --- | --- |
| **Aspect of methodological rigor** | **Definition of concept** | **How it was considered in the study** |
| **Triangulation** | Use of multiple datasets, methods, and/or investigators to address a research question | - Two methods of data collection - Two researchers in the focus groups - Multiple researchers were involved in the data analysis |
| **Data saturation** | The point at which no new themes or insights emerge from the data; conceptual categories are comprehensive and well-grounded | - Within focus-groups: field assistant ensured all interview guide topics were covered, and all participants had the opportunity to contribute - Across focus groups: saturation assessed by recurrence of categories across sessions |
| **Credibility** | Confidence in the truth of the data and how well the study captures participants experiences without omitting relevant insights. | - Multiple researchers engaged in the analysis to reduce bias - Use of illustrative quotes from a broad range of participants to support interpretations |
| **Reliability (dependability)** | Consistency and transparency of the research process; ability to track how data were coded and interpreted | - Detailed documentation of the categorization process in Microsoft Excel - Version tracking of Microsoft Excel files preserved throughout analysis - A codebook with definitions was maintained to ensure consistency across the coding process |
| **Generalization (transferability)** | Extent to which findings may be applicable to other settings, populations or contexts | - Purposeful stratification of participants based on EA-TEF characteristics and child age - Thorough description of the sample and healthcare setting provided |
| **Confirmability** | Objectivity and neutrality of the findings; the degree to which results are shaped by participants rather than researcher bias | - Use of reflexivity and self-reflection - Triangulation of data collection and analysis |
