# Supplementary material for: Parents’ experiences of condition management in children born with esophageal atresia-tracheoesophageal fistula during their early childhood
Source: Orphanet J Rare Dis. 2026 Feb 27;21:122. doi: 10.1186/s13023-026-04288-4 (PMC13040731; doi:10.1186/s13023-026-04288-4)
Supplement: Supplementary file 4 — Supplementary Material 4 [file 13023_2026_4288_MOESM4_ESM.docx]

Across data saturation:

| **Presentation of across data saturation illustrating that statements (marked with X for each participant) which build up the categories were made in all five focus groups** | | | | | | |
| --- | --- | --- | --- | --- | --- | --- |
| **Study Participants** | **Focus Group (FG)** | **Taking responsibility for the child’s health needs** | **Challenges and possibilites in understanding the child’s condition/symptoms** | **Navigating through and finding supports in health care and community-based support programs** | **Managing the child’s transition into contexts outside the home: Babysitters, daycare, and school** | **Defining the role of patient/peer-support** |
|  | FG1 | X | X | X | X | X |
|  |  | X | X | X | X | X |
|  |  | X | X | X | X | X |
|  |  | X | X | X | X | X |
|  | FG2 | X | X | X | X |  |
|  |  | X | X | X | X | X |
|  |  | X | X | X | X |  |
|  |  | X | X | X | X |  |
|  | FG3 | X | X | X | X | X |
|  |  | X | X | X | X | X |
|  |  | X | X | X | X | X |
|  |  | X | X | X | X |  |
|  | FG4 | X |  | X |  |  |
|  |  | X | X | X | X | X |
|  |  | X | X | X | X |  |
|  |  | X | X | X | X | X |
|  |  | X | X | X | X | X |
|  | FG5 | X | X | X | X |  |
|  |  | X | X | X |  | X |
|  |  | X | X | X | X | X |
|  |  | X | X | X | X |  |
|  |  | X | X | X | X | X |

Within data saturation: Each topic in the interview guide (Supplemental material 1) was addressed and all parents had the chance to contribute to the discussions (field notes).
